# Microsatellite instability (MSI-H) is associated with a high immunoscore but not with PD-L1 expression or increased survival in patients (pts.) with metastatic colorectal cancer (mCRC) treated with oxaliplatin (ox) and fluoropyrimidine (FP) with and without bevacizumab (bev): a pooled analysis of the AIO KRK 0207 and RO91 trials

**DOI:** 10.1007/s00432-021-03559-w

**Published:** 2021-03-06

**Authors:** Stefanie Noepel-Duennebacke, Hendrik Juette, Karsten Schulmann, Ulrich Graeven, Rainer Porschen, Jan Stoehlmacher, Susanna Hegewisch-Becker, Arne Raulf, Dirk Arnold, Anke Reinacher-Schick, Aandrea Tannapfel

**Affiliations:** 1 Department for Hematology, Oncology und Palliative Care, St. Josef-Hospital, Ruhr-University, Bochum, Germany; 2 Institute of Pathology, Ruhr-University Bochum, Bürkle-de-la-Camp-Platz 1, 44789 Bochum, Germany; 3 Department for Hematology and Oncology, Klinikum Hochsauerland, Meschede, Germany; 4 Department for Hematology, Oncology and Gastroenterology, Kliniken Maria-Hilf Mönchengladbach, Mönchengladbach, Germany; 5 Medical Department, Klinikum Bremen-Ost, Bremen, Germany; 6 Individuelle Krebsberatung, Bonn, Germany; 7 HOPE—Practice for Oncology, Hamburg, Germany; 8 Center for Protein Diagnostics, Bioinformatics Group, Faculty of Biology and Biotechnology, Ruhr-University, Bochum, Germany; 9 Asklepios Cancer Center, Department for Hematology, Oncology and Palliative Care, Asklepios Klinikum Altona Hamburg, Hamburg, Germany

**Keywords:** Metastatic colorectal cancer, Immunoscore, PD-L1, Microsatellite instability, Bevacizumab, Overall survival

## Abstract

**Introduction:**

In a retrospective analysis of two randomized phase III trials in mCRC patients treated first line with oxaliplatin, fluoropyrimidine with and without Bevacizumab (the AIO KRK 0207 and R091 trials) we evaluated the association of high microsatellite instability (MSI-H), immunoscore (IS) and PD-L1 expression in relation to overall survival (OS).

**Methods:**

In total, 550 samples were analysed. Immunohistochemical analysis of the MMR proteins and additionally fragment length analysis was performed, molecular examinations via allele-discriminating PCR in combination with DNA sequencing. Furthermore PD-L1 and IS were assessed.

**Results:**

MSI-H tumors were more frequent in right sided tumors (13.66% vs. 4.14%) and were correlated with mutant BRAF (*p* = 0.0032), but not with KRAS nor NRAS mutations (MT). 3.1% samples were found to be PD-L1 positive, there was no correlation of PDL1 expression with MSI-H status, but in a subgroup analysis of MSI-H tumors the percentage of PD-L1 positive tumors was higher than in MSS tumors (9.75% vs. 2.55%). 8.5% of samples showed a positive IS, MSI-H was associated with a high IS. The mean IS of the pooled population was 0.57 (SD 0.97), while the IS of MSI-H tumors was significantly higher (mean of 2.4; SD 1.4; *p* =< 0.0001).

**Discussion:**

Regarding OS in correlation with MSI-H, PD-L1 and IS status we did not find a significant difference. However, PD-L1 positive mCRC tended to exhibit a longer OS compared to PD-L1 negative cancers (28.9 vs. 22.1 months).

## Background

The development of colorectal cancer (CRC) follows distinct pathways involving microsatellite instability (MSI-H) or chromosomal instability (CIN) and is triggered by molecular mutations (BRAF, RAS, PI3K, APC, EGFR, TP53, etc.) which may offer individualized therapy strategies. Well-known positive prognostic factor for CRC is MSI-H especially in early stages (Noepel-Duennebacke et al. 2020). MSI-H patients (pts.) exhibit a superior overall survival (OS) compared to microsatellite stable (MSS; (Dienstmann et al. 2017; Klingbiel et al. 2015)). Patients with MSI-H early colon cancers are not recommended to receive adjuvant chemotherapy in UICC stage II. Furthermore, various studies have demonstrated substantial activity of checkpoint inhibition in MSI-H UICC stage IV cancers (Le et al. 2020; Overman et al. 2017, 2018). The incidence of MSI-H varies in relation to tumor stage; UICC I and II approx. 20%; UICC III 12%; UICC IV 5% (Battaglin et al. 2018) and should be tested in clinical routine.

A germline mutation (MT) of one of the DNA-mismatch-repair proteins MLH1, MSH2, MSH6, PMS2 and or deletion of EPCAM leads to deficient MMR (dMMR). Loss of a MMR-protein can be detected via immunohistochemistry (IHC) of tumor and normal tissue (Franke et al. 2018). Alternatively, fragment length analysis (FLA) via PCR from extracted tumor-DNA can reveal the characteristically MSI-H phenotype (Boland et al. 1998; Aaltonen et al. 1994). MSI-H is known as a histopathological marker to detect the Lynch-Syndrome (LS, (Boland et al. 1998)). Another reason for MSI-H, respectively, MLH1 loss is a BRAF-MT leading to methylation of the MLH1 promoter and thereby gene silencing (Deng et al. 2004). A BRAF-MT excludes LS and is characteristic for sporadic CRCs (Schmiegel et al. 2017).

MSI-H tumors can be associated with an increased immune cell infiltrate and potentially high PD-L1 expression. The level of lymphocyte invasion was described earlier within the immunoscore (IS) as a positive prognostic factor (Galon et al. 2016). However, mismatch repair deficiency leads to hypermutations that generates neoantigens. These affect immune cells mainly T-lymphocytes to invade into the tumor microenvironment to present these neoantigens via MHC to recruit more immune active cells. To eliminate this immunological unbalance, MSI-H tumors increase the expression of immunosuppressive checkpoints such as PD-1, PD-L1, and CTLA4 (Llosa et al. 2015). This immunoactivation can be measured via IS and PD-L1 expression. The IS may function as an additional biomarker for response to tumor therapy but is not yet validated. Likewise tumor mutational burden (TMB) may function as a predictor for response and prognosis, a retrospective analysis from the CALGB/SWOG80405 trial demonstrated a superior OS among tumors with high TMB (Innocenti et al. 2019).

This is a retrospective analysis of two randomized phase III trials (AIO KRK 0207, R091; (Hegewisch-Becker et al. 2015; Porschen et al. 2007)) of the association of mCRC treated first line with oxaliplatin (ox), fluoropyrimidine (FP) with and without bevacizumab (bev) and MSI-H, IS and PD-L1 expression in relation to OS.

## Materials and methods

### Patients

A total of 581 samples (201 pts. from the AIO R091 and 380 pts. from the AIO 0207 trial) were available. In 550 samples, material was sufficient and pooled for this analysis. Both studies investigated in a phase III design first-line treatment regimes in newly diagnosed mCRCs. The R091 trial compared the efficacy of CAPOX vs. FUFOX in mCRC while the AIO 0207 trial evaluated maintenance strategies (fluoropyrimidine/bev vs. bev vs. no therapy) after 24 weeks of an induction chemotherapy with combination treatment (Hegewisch-Becker et al. 2015; Porschen et al. 2007).

### Methods

**Fig. 1:**
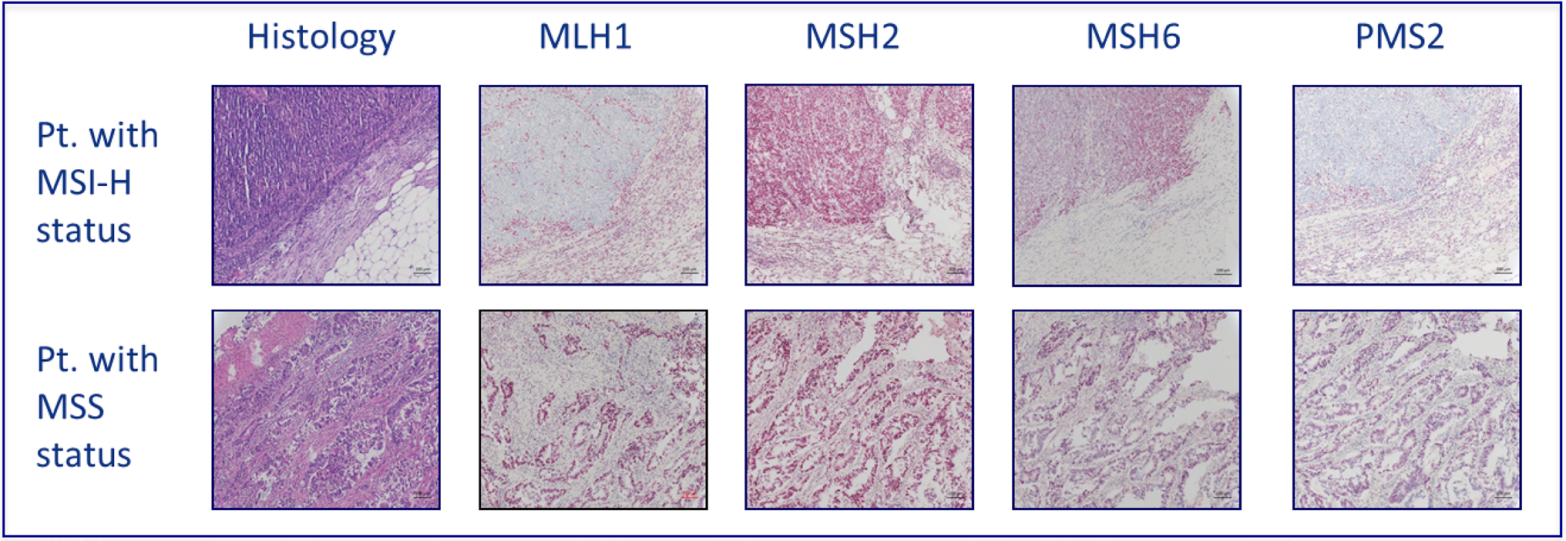
H&E staining (histology) and immunohistochemistry of examples of patients with MSI-H status (defective mismatch repair, dMMR; MLH1 and PMS2 loss) versus MSS status (proficient mismatch repair; pMMR) in mCRC pts. under oxaliplatin and fluoropyrimidine containing combination therapy

Formalin-fixed, paraffin-embedded tissue samples were available and analyzed in a central pathology. Microsatellite status (MS) was determined by IHC of DNA-mismatch repair proteins MLH1, MSH2, MSH6 and PMS2 in tumor and normal tissue. IHC was performed according to Boland (Boland et al. 1998). If at least one protein loss was detected, the tumor was classified as dMMR (Fig. 1). When a protein loss or incoherent results were noticed subsequent FLA via PCR was assessed using BAT25, BAT26, D2S123, D5S346, and D17S250 markers. MSI-H was defined if ≥ 2 out of 5 markers were found instable. The based method was, according to the literature, immunohistochemical visualization of MLH1, MSH2, MSH6 and PMS2 to define MSI-H tumors. In case of any problems of the immunohistochemistry results—or in case lesser than positive stained tumor cell nuclei—, PCR-based fragment length analysis was performed. To determine the cellular immune response, immunohistochemical stains were prepared for CD8 and CD45RO. The evaluation was performed semi-quantitatively in the tumor center as well as at the invasive margin following Galon el al. (Galon et al. 2016, Fig. 2) If the staining for CD8 or CD45RO showed an increased number of lymphocytes either in the tumor center or at the invasive margin, one point was given for that case. The resulting cumulative lymphocyte response score ranged from 0 (no response) to 4 (very strong immune response).and was afterwards converted into a binary score (0 = low = IS 0–2; 1 = high = IS 3–4). In addition, the samples were analyzed for PD-L1 expression of the tumor cells (with a 1% expression threshold, Galon et al. 2016, Fig. 3). PD-L1 expression (DAKO Mouse Anti-Human PD-L1, Clone 22C3) of the tumor cells was identified by IHC and measured in three categories: 0 (< 1% of the tumor cells), 1 (≥ 1–49% of tumor cells), and 2 (≥ 50% tumor cells). Groups 1 and 2 were counted as PD-L1 positive cases. We considered only membranous staining of any intensity to be relevant. Tumor tissue from primary tumor specimen (*n* = 395) and from resected metastases (*n* = 166) were analyzed. Mutation analysis of KRAS, NRAS and BRAF were performed after extraction the genomic DNA from microdissected paraffin-embedded tissue with allele-discriminating PCR in combination with DNA sequencing. Statistical analysis was performed using log rank test and Cox regression.

**Fig. 2:**
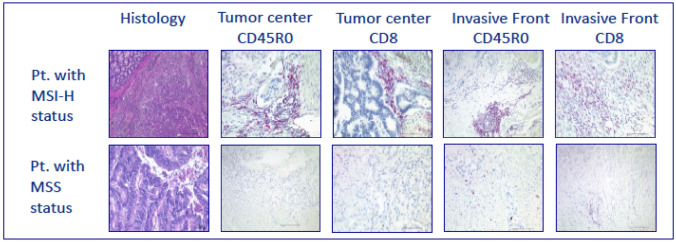
Immunoscore according to Galon et al. (2016) in MSI-H and MSS mCRCs; tumor center vs. invasive front

**Fig. 3:**
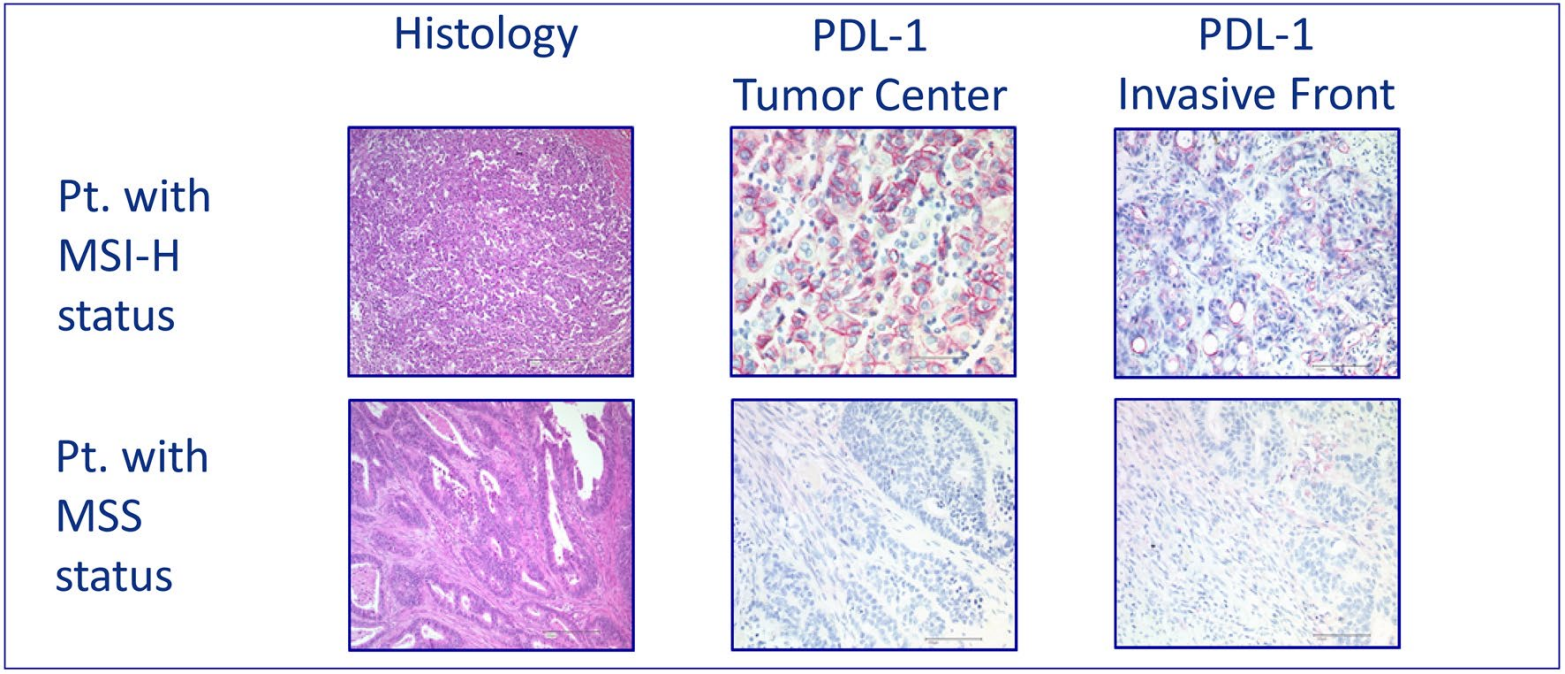
Examples of MSI-H versus MSS in mCRC, HE-staining (histology), IHC of PD-L1 in tumor center and invasive front

## Results

**Fig. 4:**
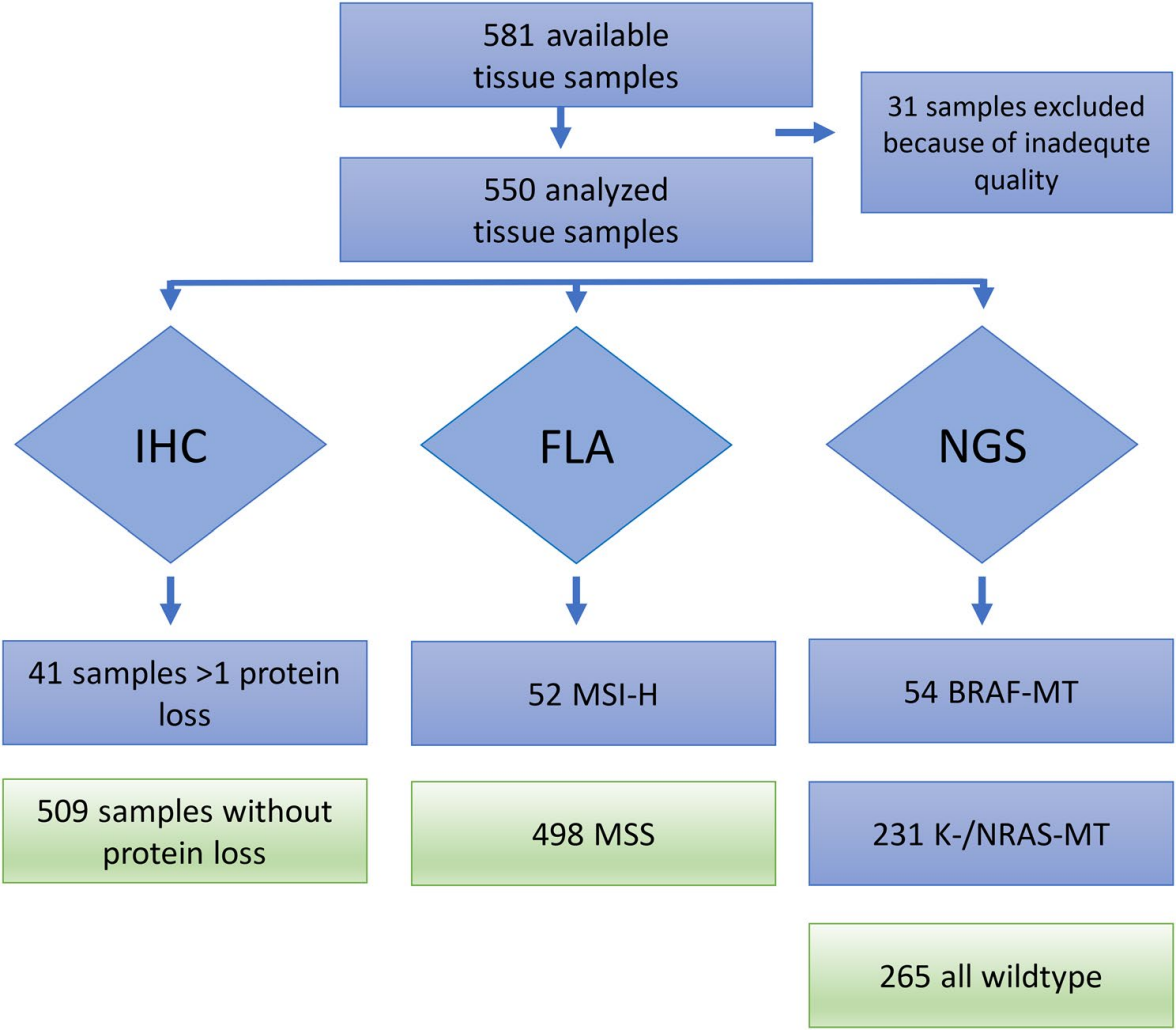
Sample distribution and histopathological analysis; immunohistochemistry (IHC), fragment length analysis (FLA), next-generation sequencing (NGS), mutation (MT)

Out of both studies, finally 581 tissue samples were available and finally 550 samples analyzed. 31 samples were excluded because of inadequate quality (Fig. 4).

### Patient´s characteristics

The median age was 63.3 years (yrs.) and did not differ between MSI-H and MSS pts. Compromising gender more females displayed MSI-H tumors (MSI-H: 19/204; 9.31%) as men (MSI-H: 22/346; 6.35%). Regarding MSI-H and localization of the primary tumor 14/338 (4.14%) mCRCs were left- and 22/161 (13.66%) were right-sided, showing a significant difference. There was no difference in age, grading (G) and lymph node status (Table 1).

**Table 1:** Baseline characteristics according to microsatellite status (MSI-H vs. MSS)

	mCRC pts. with MSI-H status (*n* = 41)	mCRC pts. with MSS status (*n* = 509)	All (*n* = 550)
Age Median (years)	62.27	63.70	63.62
Gender Female Male	19 (46.34%) 22 (53.66%)	185 (36.35%) 324 (63.65%)	204 (37.09%) 346 (62.91%)
Grading G1 G2 G3 G4 Not known	35 (85.37%) 0 (0.00%) 22 (53.66%) 12 (29.27%) 1 (2.44%) 6 (14.63%)	415 (81.53%) 3 (0.59%) 291 (57.17%) 120 (23.58%) 1 (0.20%) 94 (18.47%)	450 (81.82%) 3 (0.55%) 313 (56.91%) 132 (24.00%) 2 (0.36%) 100 (18.18%)
Localisation of primary tumor Left-sided Right-sided Not known	36 (87.80%) 14 (34.15%) 22 (53.66%) 5 (12.20%)	463 (90.96%) 324 (63.65%) 139 (27.31%) 46 (9.04%)	499 (90.73%) 338 (61.45%) 161 (29.27%) 51 (9.27%)
Node N0 N1 N2 Not known	38 (92.68%) 7 (17.07%) 11 (26.83%) 19 (46.34%) 3 (7.32%)	466 (91.55%) 80 (15.72%) 128 (25.15%) 247 (48.53%) 43 (8.45%)	504 (91.64%) 87 (15.82%) 139 (25.27%) 266 (48.36%) 46 (8.54%)

### Histopathological analysis

In total of 550 tissue probes from both studies (AIO KRK 0207 and AIO KRK R091) were available and analyzed concerning MS. All tumors, defined as MSHI-high were retested using FLA. FLA was also performed, if immunohistochemistry was impossible to assess or even unclear. IS analogues to Galon et al. and PD-L1 expression (Galon et al. 2016).

### Frequency of high microsatellite instability and mutation analysis

Via IHC 52/550 (9.5%) at least one missing MMR protein was detected. All were subjected to FLA, 41/550 (7.4%) cases displayed MSI-H. K- and N-RAS MT were discovered in 231/550 (42%) of mCRCs and BRAF (V600E) MT were found in 54/550 (9.8%). In 41 MSI-H tumors, 9 BRAF MT were found (21.9%) and 18 K- and N-RAS MT (43.9%), 14/41 MSI-H tumor were all wild type. In MSS tumors (*n* = 509), 213 K- and N-RAS MT (41.8%) and 45 BRAF-MT (8.8%) were observable. MSI-H correlated with mutant BRAF (*p* = 0.0032), but not with KRAS nor NRAS-MT (Table 2).

**Table 2:** Frequency of RAS- and BRAF-mutation (MT) of mCRCs according to microsatellite status (MS) MSI-H vs. MSS

	mCRC pts. with MSI-H status (*n* = 41)	mCRC pts. with MSS status (*n* = 509)	All (*n* = 550)
KRAS (Exon 2/3/4)	18	194	212
NRAS (Exon 2/3/4)	0	19	19
BRAF V600	9	45	54

### PD-L1 expression according to microsatellite status

In 550 samples, we found *n* = 17 (3.1%) pts. with positive expression of PD-L1 in tumor cells and 533 (96.9%) with a negative PD-L1 score (Table 3, Fig. 3). Only four of these were MSI-H. There was no correlation of PD-L1 expression with MSI-H, but in subgroup analysis, in MSI-H tumors, the percentage of PD-L1-positive tumors was higher than in MSS tumors (9.8% vs. 2.6%).

**Table 3:** PD-L1 expression and immunoscore in correlation to MS (MSI-H vs. MSS)

	All	MSI-H (%)	MSS (%)
	550	41 (7.5)	509 (92.5)
PD-L1 negative	533	37 (6.9)	496 (93.1)
PD-L1 positive	17	4 (23.5)	13 (76.5)
Immunoscore low	503	15 (3)	488 (97)
Immunoscore high	47	26 (55.3)	21 (44.7)

### IS according to MS-status

47/550 (8.5%) samples showed a positive IS according to Galon et al., (Fig. 2, Galon et al. 2016). Among 47 IS high tumors, 26 were MSI-H (55.3%) and 21 were MSS (44.7%, Table 3), which illustrates a trend for higher IS among MSI-H tumors. The mean IS of the population was 0.57 (SD 0.97), while the IS of MSI-H tumors was significantly higher (mean of 2.4; SD 1.4; *p* =< 0.0001).

### Overall survival

**Fig 5:**
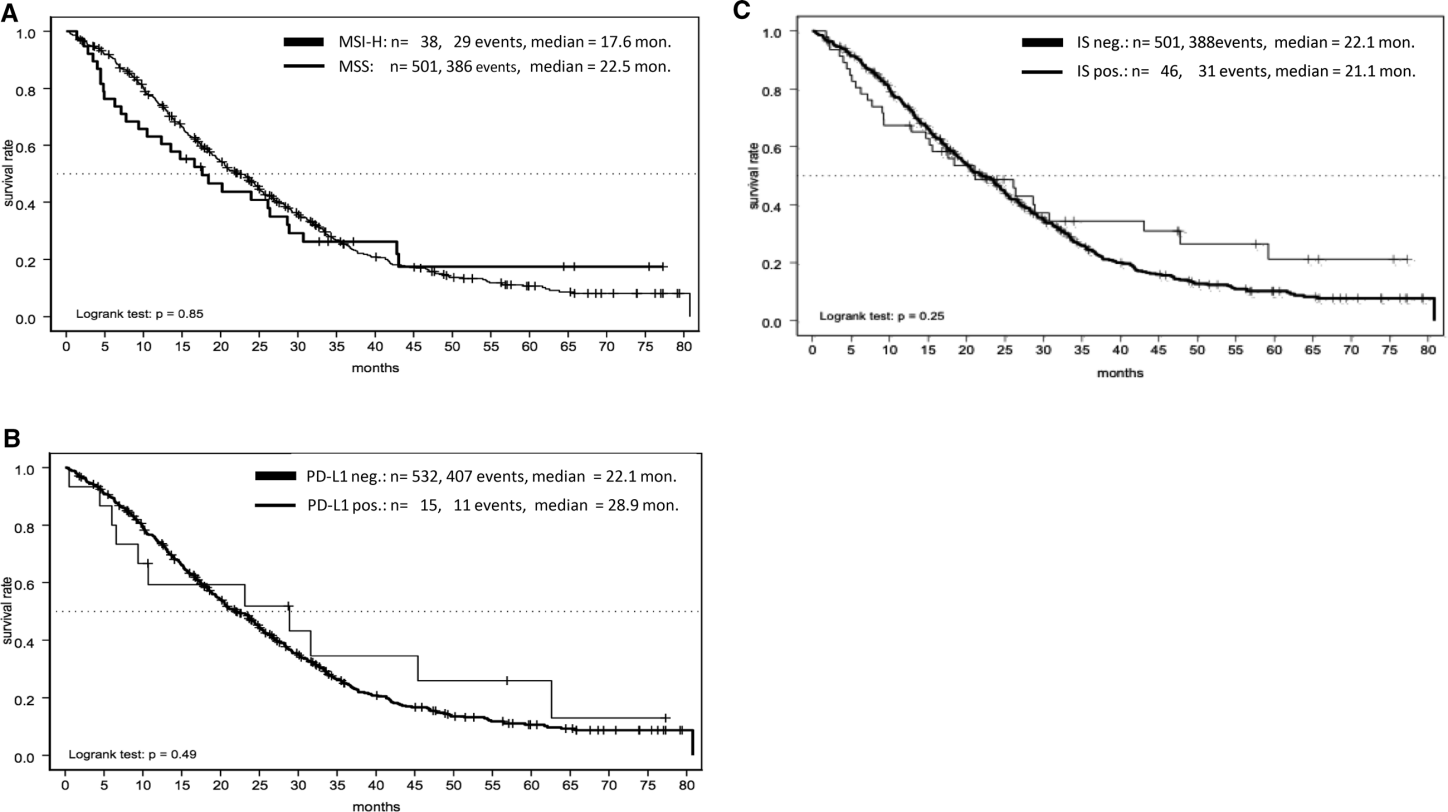
**a**–**c** OS in correlation to microsatellite status (MSI-H vs. MSS, **a**), PD-L1 expression (**b**) and immunoscore (IS, **c**)

Median OS in MSI-H mCRC was 17.6 months and in MSS pts. 22.5 months (*p* = 0.85, Fig. 5a). Comparing MSI-H and MSS mCRC in this pooled analysis, we did not find a significant difference in these groups in relation to OS. PD-L1-negative pts. median OS was 22.1 months and PD-L1-positive pts. 28.9 months (*p* = 0.49), which did not reach significance level (Fig. 5b). There was no difference in the median OS regarding IS-negative vs. -positive pts. (21.1 months vs. 22.1 months; *p* = 0.25, Fig. 5c).

## Discussion

In this pooled analysis of two randomized phase III trials (AIO KRK 0207, R091; Hegewisch-Becker et al. 2015, Porschen et al. 2007) of newly diagnosed mCRC treated first line with ox, FP with and without bev, we investigated the prognostic value of MSI-H, IS and PD-L1 expression and their correlation. MS in mCRC correlated with a positive IS, but not with PD-L1 expression, none of these immune markers seemed prognostic for OS in mCRCs.

### Frequency of MSI-H, K-/NRAS and BRAF-MT in mCRCs

The incidence of MSI-H tumors among 550 analyzed samples was 9.5% via IHC and 7.4% via FLA, which is consistent to recently published data (Innocenti et al. 2019). The divergence of both methods results from its different techniques (Kawakami et al. 2015). 42% tissue samples harbored a K- or NRAS-MT and BRAF-MT were found in 9.81%. MSI-H correlated with BRAF-MT (21.95% vs. 8.84%; *p* = 0.0032), which is likewise coherent to known data (Yaeger et al. 2018). Mutation of BRAF leads via CpG island methylation to gene silencing, affecting most frequently the MLH1-promotor, which can cause an MSI-H phenotype (Deng et al. 2004). MLH1 promoter methylation analysis should be done in the context of MLH1 loss to distinguish between hereditary MLH1 loss because of a gene MT and sporadic acquired MLH1 loss caused by BRAF-MT (Newton et al. 2014).

### PD-L1 expression and IS in mCRCs

We found a low frequency of PD-L1 expression in this pooled cohort, only 3.1% (Table 3, Fig. 3). Via subgroup analysis a higher percentage of PD-L1-positive tumors was detected among MSI-H tumors than in MSS tumors (9.75% vs. 2.55%). MSI-H tumors accumulate MT like mismatch-base-paring, deletions and insertions like frameshift-MT, and, therefore induce neoantigens (Franke et al. 2018). These neoantigens lead to a higher density of tumor infiltrating lymphocytes, which can be quantified using the IS (Galon et al. 2016). In our cohort, 47 IS high samples were detected, 55.3% were MSI-H tumors (Fig. 2). The mean IS of the whole population was 0.57 (SD 0.97), while the IS of MSI-H tumors was significantly higher (mean of 2.4; SD 1.4; *p* = < 0.0001), confirming the well-known observation that MSI-H tumors exhibit higher IS.

### Molecular markers and survival

Regarding OS and MS, PD-L1 expression and IS, there was no significant difference between these markers under a first-line treatment with ox/FP with and without bev (Fig. 5a–c). The prognostic value of MSI-H in mCRC still remains uncertain. Recently published data from CALGB/SWOG 80405 analyzed the prognostic value of MSI-H under first-line treatment with chemotherapy either with bev or EGFR-antibody cetuximab (Innocenti et al. 2019). Comparing MSI-H (*n* = 52/827, 6%) and MSS tumors, MSI-H was a negative prognostic factor concerning OS (*p* = 0.087). In our analysis, median OS among MSI-H pts. was shorter compared to MSS pts., but did not reach significance level (17.6 vs. 22.5 months; *p* = 0.85, Fig. 5a) potentially due to lower numbers. In addition, Innocenti et al. found a significant longer OS of MSI-H tumors when treated with bev compared to cetuximab (HR 0.13; 95% CI 0.06–0.3; *p* < 0.001) which lead the authors to the hypothesis that MSI-H might be predictive for anti-VEGF-treatment (Innocenti et al. 2019). These findings are consistent with adjuvant treatment studies adding (placebo-controlled) bev to FOLFOX in UICC stage II/III MSI-H tumors (Pogue-Geile et al. 2013) but this regimen did not find its way into clinical routine because of negative overall results.

17 tumor tissue samples harbored a high (> 1%) PD-L1 score, only 4 of these were MSI-H (Table 3), there was no correlation between OS and PD-L1 score (*p* = 0.49, Fig. 5b). Histopathological MSI-H tumors can be associated with an increased immune cell infiltrate and potentially high PD-L1 expression and higher IS (Galon et al. 2016). PD-L1 expression and IS have been shown to be a predictive biomarker for immunotherapy in various cancers. Data investigating PD-1 inhibitor Nivolumab in MSI-H mCRC presented an ORR 31.1%, in a median follow-up from 12.0 months, 68.9% of pts. achieved a disease-free survival (DFS) for ≥ 12 weeks (Overman et al. 2017). Among double immunotherapy (Nivolumab plus Ipilimumab), the ORR increased up to 55% within a median follow-up of 13.4 months, 80% had a DFS for ≥ 12 weeks (Overman et al. 2018). PD-1 monotherapy in further lines with Pembrolizumab in MSI-H mCRCs lead to an ORR of 33% and an impressive median OS of 31.4 months in pts. treated ≥ 2 lines, OS of > 1 prior therapy was not reached at publication date (Le et al. 2020). The Keynote 177 trial investigated first-line treatment of MSI-H mCRCs with Pembrolizumab and confirmed a n ORR of 43.8% leading to a PFS of 16.5 months vs. 8.2 months in the chemotherapy group (HR 0.6; *p* = 0.0002, André et al. 2020) setting the standard for first-line treatment of MSI-H mCRC. However, PD-L1 expression and validated biomarkers in mCRCs (e.g., BRAF, RAS) nor their correlation were found to be predictive for the response to PD-1 pathway blocking antibodies in those analyses. The reason for the discordance between response rates among checkpoint inhibitors and PD-L1 expression may due to the fact that the PD-L1 expression is a dynamic marker of the cell surface which depends from its interaction with its environment and my change during therapy (Franke et al. 2018). These facts suggest that PD-L1 might not be useful as a predictive marker. To establish additional biomarkers, Mlecnik et al. validated the prognostic impact of tumor infiltrating immune cells (summarized within an immunoscore) in CRCs and its relation to MS (Mlecnik et al. 2016). First, they pointed out, that MSI-H does not necessarily lead to a high IS; in our analysis, we detected 47/550 pts. with a high IS, in subgroup analysis concerning MS 26/41 were MSI-H (55.3%) whereas 21/41 (44.7%) were MSS, which is consistent to data from Mlecnik. In our cohort, the IS of MSI-H tumors were significantly higher (mean of 2.4; SD 1.4; *p* = < 0.0001). Regarding OS, there was no difference between IS high and low (Fig. 5c). These findings differ from previous published investigations. Mlecnik et al. detected a better prognosis for pts. with high IS (concerning DFS: HR = 6.35; OS: HR = 3.96) compared to low IS. Furthermore, the density of tumor infiltrating immune cells improved OS and DFS even despite microsatellite status (Mlecnik et al. 2016). Out of their findings, Mlecnik suggested, regardless to MS, only mCRCs with a high IS may benefit from checkpoint inhibitors. Our cohort comprised 47 pts. (8.5%) with a high IS so its power to detect significant differences was limited. Data concerning the predictive role of IS are rare and only based on retrospectives subgroup analysis, and therefore, of lacking evidence. Furthermore, the first-line treatment of this pooled cohort comprised of different chemotherapy regimen and maintenance strategies, but these were not found to show any impact on survival in correlation with the analyzed markers. The role of IS remains unclear in mCRCs and needs to be validated in a prospective trail design, ideally combined with known prognostic (BRAF, RAS), molecular makers (PD-L1 expression) and tumor mutational burden (TMB). TMB reflects the amount of acquired tumor MT, MSI-H and other MT (i.e., POLE) can lead a high TMB through accumulation of MT (Silberman and Steiner 2019) and consequently lead to a high IS, which can potentially lead to a higher sensitivity to immunotherapy. However, until now no predictive marker regarding this question is known, maybe TMB reveals predictive impact concerning immunotherapy in mCRCs.

Limitations of this pooled analysis concern various aspects of the two studies. Our analysis pooled data from the AIO KRK 0207 and AIO KRK R091 trial, both first line trials of patients with mCRC. However, there were substantial differences regarding the exact treatment protocol, the length of treatment and time when the trial was conducted and published (and thus, also the impact of further line treatment on survival) as well as primary endpoints. Both trials reported the OS of the included population, which we considered in our pooled evaluation. In detail, the R091 trial compared the efficacy of CAPOX vs. FUFOX as first-line treatment and was conducted between 2002 and 2004 and was published in 2007 (Porschen et al. 2007). Mean treatment duration was 20.6 and 21.7 weeks. PFS was the primary endpoint, which did not differ significantly between the arms (CAPOX: 7.1 months vs. FUFOX: 8.0 months; *p* = 0.177). OS as secondary endpoint was 17.3 months for all pts. (CAPOX: 16.8 months vs. FUFOX: 18.8 months; *p* = 0.26). Molecular markers were not reported during the first publication due to the time of initiation. AIO-KRK 0207 used a 24-week induction chemotherapy (FP/Ox/Bev) to evaluate maintenance strategies (FP/bev vs. bev vs. no therapy) in pts. achieving at least stable disease and was conducted between 2009 and 2013 (Hegewisch-Becker et al. 2015). The primary endpoint was the time to failure of strategy, defined as time from maintenance (randomization) to second progression, i.e. the beginning of second line treatment, no further treatment or death. For all pts., time to failure of strategy was 6.5 months (FP/bev: 6.9 months, bev: 6.1 months, no therapy: 6.4 months). 36% of randomized pts. underwent re-induction after maintenance. Survival analysis considered PFS and OS from enrollment and randomization. Our analysis only considered OS from enrollment. In addition, the AIO 0207 trial included anti-VEGF treatment (Porschen et al. 2007). Anti-VEGF treatment was the main difference in treatment protocol as well as further line treatment and may be a bias concerning survival analysis because anti-VEGF-treatment prolongs PFS. However, it has not prolonged OS. In addition, as mentioned above, Innocenti et al. detected a longer OS among MSI-H tumors treated with bevacizumab (Innocenti et al. 2019). However, in this small sample sizes of MSI-H tumors (7.4%), the statistical influence, respectively, impact of anti-VEGF-therapy remains unclear.

In summary, both trials differ somewhat concerning their design, primary endpoint and treatment strategies. However, they were very similar regarding the first line treatment protocol (FP and Ox), pts. cohort with a similar median age (AIO KRK R091: 65 yrs; AIO 0207: 64 yrs.) and the impact of survival (OS; Hegewisch-Becker et al. 2015, Porschen et al. 2007). We used these survival data (primarily OS) for our evaluation. Main focus was molecular markers and their correlation in newly diagnosed mCRC. Main focus was molecular markers and their correlation in newly diagnosed mCRC, precisely the incidence and association of MSI-H, PD-L1 and IS. Decisive results were the characteristics of MSI-H mCRCs: MSI-H tumors had a significantly higher mean IS (Table 3) as well as PD-L1 expression. These molecular markers are independent from therapy strategies and trial designs, but serve to characterize tumor tissue to enable personalized therapy strategies. Nowadays, molecular markers predict outcomes and treatment. Our survival analysis as an additional point of this pooled analysis detected no significant difference in relation to molecular markers with exception of MSI-H tumors. Our results should not primarily serve for treatment recommendations, but state a trend for further analyses of molecular markers and their correlation to establish personalized therapy options.
